# Assessing the Impact of All-Trans Retinoic Acid (ATRA)- and Arsenic Trioxide (ATO)-Based Therapy in Pediatric Acute Promyelocytic Leukemia: A Single-Center Study

**DOI:** 10.7759/cureus.89081

**Published:** 2025-07-30

**Authors:** Reshma Roshan, Mubashir H Shah, Sherook J, Aidel F Parry, Javid R Bhat

**Affiliations:** 1 Clinical Hematology, Sher-i-Kashmir Institute of Medical Sciences (SKIMS), Srinagar, IND; 2 Department of Pediatrics, Government Medical College Srinagar, Srinagar, IND

**Keywords:** acute myeloid leukemia (aml), high-risk patients, induction therapy, pediatric patients, pml-rara fusion gene, risk stratification, treatment regimen

## Abstract

Background: Acute promyelocytic leukemia (APL) is a subtype of acute myeloid leukemia (AML) characterized by the t(15;17) translocation, leading to the PML-RARA fusion gene. While treatable, APL presents significant challenges, particularly in resource-constrained settings where delays in diagnosis and access to specialized care may impact outcomes. This study aims to describe the clinical presentation, treatment outcomes, and survival data for pediatric APL patients.

Methods: This observational, retrospective, single-center study was conducted at Sher-i-Kashmir Institute of Medical Sciences (SKIMS), Srinagar, spanning for a period of six years. The study included 20 pediatric patients diagnosed with APL. Laboratory profiles, treatment regimens, and complications were analyzed. Risk stratification was done using modified Sanz criteria, and patients were treated according to the APL0406 and APML4 protocols. Overall survival (OS) and Progression-free survival (PFS) were calculated over a median follow-up period of three years.

Results: Median OS was 88 months (95% CI= 79.612 to 97.188). The mean haemoglobin levels were 6.51 ± 1.82 mg/dL and patients had high PML-RARA transcript levels (5175.95 ± 3039.37). Common symptoms included mucocutaneous bleeding (19, 95%) and gum bleeding (10, 50%). Induction with all-trans retinoic acid (ATRA) and arsenic trioxide (ATO) lasted a mean of 40.9 days. Febrile neutropenia (16, 80%) and differentiation syndrome (14, 70%) were frequent complications. One patient (1, 5%) died during induction.

Conclusion: This study reinforces the effectiveness of ATRA-ATO-based regimens in managing paediatric APL. However, induction-related complications such as febrile neutropenia, transaminitis, and QTc prolongation highlight the need for vigilant monitoring and robust supportive care. Timely diagnosis and early initiation of therapy remain key to improving outcomes.

## Introduction

Acute promyelocytic leukemia (APL), a distinct subtype of acute myeloid leukemia (AML), accounts for approximately 5-12% of AML cases globally, with marked geographic variation in incidence. Most of the cases were reported in Caucasians (57.34%), followed by Hispanics (22.75%), Blacks (10.6%), and Asian/Pacific Islanders (8.4%) [1,2]. In the pediatric population, acute lymphocytic leukemia (ALL) constitutes the majority (76%), while AML represents 15-20% of childhood leukemia cases globally. Despite being less frequent, AML is responsible for up to 30% of pediatric leukemia-related deaths, highlighting its clinical significance. De novo APL, a biologically and clinically unique subtype of AML, constitutes about 5-10% of childhood AML cases in the United States [3]. Characterized by the hallmark translocation t(15;17)(q24.1;q21.2) leading to the PML-RARA fusion gene, APL exhibits unique clinical and biological behaviour attributed to its disruptive retinoic acid signalling [4]. The disease often presents as a medical emergency due to a high risk of life-threatening hemorrhage, frequently attributed to an underlying coagulopathy involving disseminated intravascular coagulation (DIC) and hyperfibrinolysis, which result in rapid depletion of clotting factors and platelets [5].

The diagnosis of APL has been improved by advances in molecular diagnostics and therapeutic interventions. Morphologically, APL is categorized into two subtypes: the classical hypergranular form comprising one-third of all cases, and the microgranular variant, which accounts for approximately 15-20% of cases and often poses greater diagnostic challenges [6,7]. The management of APL was primarily based on traditional chemotherapy regimens such as anthracyclins like daunorubicin combined with cytarabine, similar to those used for other subtypes of AML. These regimens achieved response rates ranging from 55% to 88%; however, the outcomes were considered suboptimal due to high relapse rates and treatment-related toxicity [8]. The introduction of all-trans retinoic acid (ATRA) as a differentiation agent, followed by arsenic trioxide (ATO), has transformed APL from a highly fatal disease into one of the most curable hematologic malignancies [9,10]. The therapeutic success of ATRA and ATO in APL lies in their ability to target the underlying molecular defect, the PML-RARA fusion protein. ATRA binds to the altered RARα portion of the fusion protein, restoring transcriptional activity and promoting the differentiation of malignant promyelocytes into mature granulocytes. ATO, on the other hand, targets the PML component, inducing degradation of the PML-RARA oncoprotein through oxidative stress and enhancing apoptosis. This synergistic approach eradicates the leukemic clone by promoting both differentiation and programmed cell death [11]. The AIDA 0493 protocol, combining ATRA with ATO, has yielded complete remission (CR) rates exceeding 80%, with 68.9% event-free survival observed at 12 years in the majority of patients. Despite these advances, early mortality remains a major challenge, particularly in resource-constrained settings, due to delays in diagnosis and initiation of treatment [12].

Data from India remains limited, highlighting the importance of region-specific studies. North India, with its varied demographic and geographic characteristics, has relatively sparse data on APL. A study in this area and region can help bridge the gap in existing literature and enhance understanding of the clinical presentation, treatment outcomes, and disease patterns of APL in North India. This study aims to describe the demographic, clinical, hematologic, and treatment-related characteristics of APL patients, with a focus on treatment outcomes. The findings are expected to inform regionally relevant management strategies for APL.

## Materials and methods

Study design

This was an observational, retrospective, single-center study conducted at the Department of Clinical Hematology and Bone Marrow Transplant, Sher-i-Kashmir Institute of Medical Sciences (SKIMS), Srinagar, Jammu and Kashmir, India. The study period spanned from January 2017 to December 2023.

Objectives

The primary aim was to analyze the clinical and laboratory profile of patients diagnosed with APL, along with their treatment response and treatment-related complications.

Participants

All pediatric (<18 years of age) patients diagnosed with APL during the study period and registered under the Department of Clinical Hematology were screened for eligibility. The inclusion criteria comprised all patients of any age or gender who were diagnosed with APL based on immunophenotyping, cytogenetic confirmation of the t(15;17) (q24.1;q21.2) translocation, and/or detection of the PML-RARA fusion transcript by molecular testing. Patients were excluded if they were diagnosed with bi-phenotypic leukemia involving both myeloid and lymphoid lineages, acute undifferentiated leukemia, or other subtypes of AML as diagnosed by flow cytometry or morphology.

**Figure 1:**
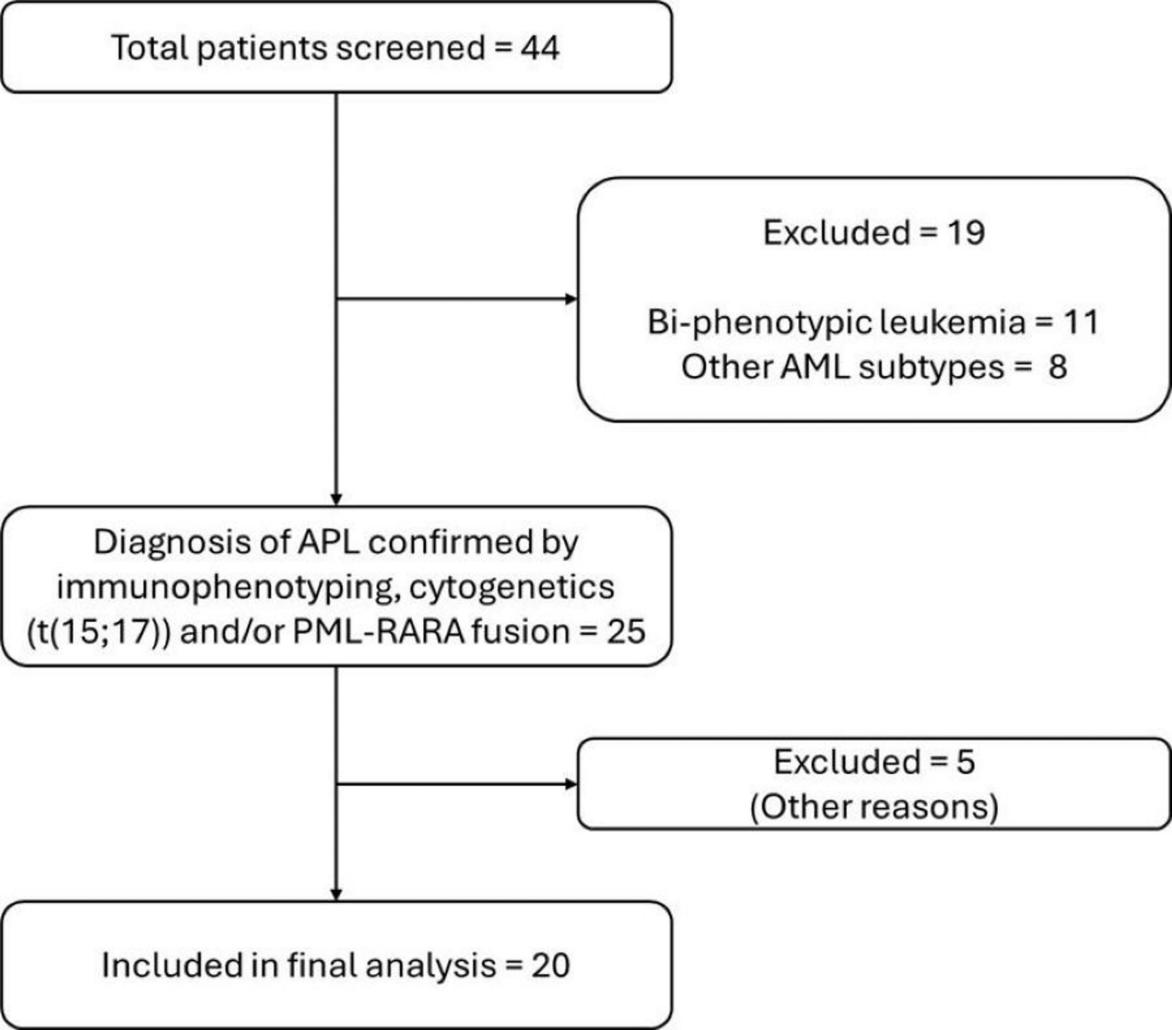
Consolidated Standards of Reporting Trials (CONSORT) Flow Diagram of Pediatric Patients Diagnosed With Acute Promyelocytic Leukemia (APL) AML: acute myeloid leukemia

Data sources and measurement

Clinical data were retrieved from patient records and departmental databases. Diagnostic confirmation was made through peripheral smear and bone marrow morphology, supported by flow cytometry markers (CD13+, CD33+, HLA-DR−), cytogenetic analysis for t(15;17), and molecular testing for PML-RARA using reverse transcriptase polymerase chain reaction (RT-PCR). Coagulation parameters including prothrombin time (PT), activated partial thromboplastin time (APTT), fibrinogen, and D-dimers were used to assess DIC at baseline. Patients were stratified using the modified Sanz criteria [13]. Initially, they were categorized into low-, intermediate-, and high-risk groups based on their presenting white blood cell (WBC) and platelet counts. However, for treatment purposes, patients were further grouped into high-risk (WBC >10,000/μL) and non-high-risk (WBC ≤10,000/μL, combining low and intermediate risk groups)

Treatment protocol

Non-high-risk patients were treated according to the APL0406 protocol [14], which included oral ATRA at a dose of 45 mg/m²/day in two divided doses, along with intravenous ATO at a dose of 0.15 mg/kg/day. High-risk patients were managed according to the APML4 protocol, which included ATRA, ATO, and anthracycline-based chemotherapy using idarubicin (12 mg/m²) or daunorubicin (60 mg/m²) administered on days two, four, six, and eight. Induction therapy was continued until hematologic CR or for a maximum of 60 days. Bone marrow aspiration was performed at the end of induction to assess remission, and molecular monitoring for minimal residual disease (MRD) was conducted using quantitative polymerase chain reaction (PCR).

Supportive care included prophylactic antibiotics and antifungals, platelet transfusions to maintain platelet counts above 30×10⁹/L (or 50×10⁹/L in bleeding patients), and packed red blood cells to maintain haemoglobin above 8 g/dL. Cryoprecipitate and fresh frozen plasma were administered in cases of hypofibrinogenemia or deranged coagulation profiles. Patients were closely monitored for complications such as differentiation syndrome, febrile neutropenia, transaminitis, and corrected QT interval (QTc) prolongation.

Statistical methods

Descriptive analyses were used to summarize patient demographics, clinical features, laboratory values, and treatment outcomes. Qualitative variables were reported as frequencies and percentages, while quantitative variables were expressed as mean ± standard deviation or median with interquartile ranges, depending on distribution. Survival analysis was performed to assess overall survival (OS) and progression-free survival (PFS). Statistical analyses were performed using SPSS version 22.0 (IBM Corp., Armonk, NY, USA).

Ethical considerations

The study was approved by the Institutional Ethics Committee of SKIMS on February 8, 2023 (IEC/SKIMS Protocol No. 307/2022). Given the retrospective nature of part of the study, informed consent was waived. The study was conducted in accordance with the ethical principles outlined in the Declaration of Helsinki (2024).

## Results

A total of 20 pediatric patients were diagnosed with APL from January 2017 to December 2023. The mean duration of symptoms prior to presentation was 9.35 ± 5.16 days. Laboratory investigations revealed a mean hemoglobin level of 6.51 ± 1.82 mg/dL, indicating severe anemia in most patients (<8 g/dL: severe anemia; Pediatric and Adolescent Nutrition Society of the Indian Academy of Pediatrics) (Table 1) [15].

**Table 1:** Demographic, Clinical, and Key Laboratory Profile of Pediatric Acute Promyelocytic Leukemia (APL) Patients (N = 20) PML-RARA by QPCR: Quantitative PCR for PML-RARA fusion gene; Promyelocytes in Bone Marrow Aspiration and Peripheral Blood Film: Percentage of promyelocytes in bone marrow and peripheral blood; Fibrinogen, D-Dimer, and Coagulation Profile: Indicators of disseminated intravascular coagulation (DIC); APTT: activated partial thromboplastin time; Bleeding PR: bleeding per rectum; SOB: shortness of breath; Lymphadenopathy (LNE): enlargement of lymph nodes; LDH: lactate dehydrogenase; INR: international normalised ratio

Parameter (Mean ± SD)	baseline Value
Age (Years)	11.4 ± 5.27
Duration of symptoms (Days)	9.35 ± 5.16
Hemoglobin (mg/dL)	6.51 ± 1.82
Total Count (×10⁹/L)	4.28 ± 3.47
Neutrophils (%)	25.38 ± 21.43
Lymphocytes (%)	44.25 ± 21.39
Monocytes (%)	29.81 ± 24.32
Promyelocytes In Bone Marrow Aspiration (%)	84.85 ± 12.90
Promyelocytes In Peripheral Blood Film (%)	43.85 ± 27.63
PML-RARA by QPCR (Ratio %)	5175.95 ± 3039.37
Platelet Count (×10⁹/L)	17.60 ± 19.10
Prothrombin time (Seconds)	21.50 ± 26.74
INR	1.76 ± 2.24
APTT (Seconds)	41.86 ± 38.37
Fibrinogen (G/L)	1.68 ± 0.55
D-Dimer (Ng/Ml)	4516.75 ± 3508.25
LDH (U/L)	351.65 ± 98.89
Gender, N (%)	
Female	12 (60%)
Male	8 (40%)
Diabetes, N (%)	
Yes	1 (5%)
No	19 (95%)
Clinical Presentation, N (%)	
Fever	14 (70.0%)
Bleeding At Presentation	17 (85.0%)
Mucocutaneous	19 (95.0%)
Gum Bleeding	10 (50.0%)
Epistaxis	1 (5.0%)
Bleeding PR	1 (5.0%)
Heavy Menstrual Bleeding (HMB)	4 (20.0%)
Purpura/Ecchymosis	10 (50.0%)
Breathlessness/Sob	1 (5.0%)
General Weakness	9 (45.0%)
Pallor	19 (95.0%)
Lymphadenopathy (LNE)	1 (5.0%)

The PML-RARA fusion transcript was markedly elevated (5175.95 ± 3039.37%), confirming the molecular diagnosis of APL. Coagulation abnormalities were common, including prolonged PT, APTT, elevated international normalised ratio (INR) and D-dimer, and low fibrinogen levels, indicating DIC. Clinically, most patients had mucocutaneous symptoms (19, 95%), pallor (19, 95%), and bleeding (17, 85%). Lactate dehydrogenase (LDH) was elevated (351.65 ± 98.89 U/L), suggesting high cell turnover.

The mean duration of induction therapy using ATRA + daunorubicin, As2O3 or daunorubicin + cytosar was 40.90 ± 5.46 days. The average ATRA dose was 50.50 ± 24.38 mg/m²/day, and ATO dose was 6.23 ± 3.18 mg. Hydroxyurea was used in 17 (85%) patients, with a mean dose of 1.77 ± 0.87 g/day for 5.82 ± 1.19 weeks. The most common corticosteroid used was dexamethasone 8 mg (Table 2).

**Table 2:** Dosage Regimen During Induction Phase (N = 20) ATRA: all-trans retinoic acid; ATO: arsenic trioxide; DEX: dexamethasone

Parameter	Value
Duration of Induction (days, Mean ± SD)	40.90 ± 5.46
ATRA Dose (mg/m²/day, Mean ± SD)	50.50 ± 24.38
ATO Dose (mg, Mean ± SD)	6.23 ± 3.18
Dexamethasone (MG, MEAN ± SD)	5 ± 2.58
Dexamethasone, N (%)	13 (65%)
Prednisolone 30 mg, n (%)	1 (5%)
Use of Hydroxyurea – Yes, n (%)	17 (85%)
Use of Hydroxyurea – No, n (%)	3 (15%)
Hydroxyurea Dose (g/day, Mean ± SD)	1.77 ± 0.87
Duration of Hydroxyurea (weeks, Mean ± SD)	5.82 ± 1.19

During induction, febrile neutropenia occurred in 16 (80%) patients, while differentiation syndrome (DS) was seen in 14 (70%) patients, Grade II in six (30%) and Grade III in eight (40%) patients. Papilledema was observed in five (25%) patients. Other complications, though less frequent, reflected the multisystem involvement commonly seen during APL treatment (Table 3).

**Table 3:** Complications Observed During Treatment (N = 20) QTc: corrected QT interval

Parameter, N (%)	Value
Febrile Neutropenia	16 (80%)
Transaminitis	6 (30%)
Hyperbilirubinemia	2 (10%)
QTc Prolongation	5 (25%)
Infections	2 (10%)
Pneumonia	3 (15%)
Type 1 Respiratory Failure	3 (15%)
Papilledema	5 (25%)
Papilledema-NO	13 (65%)
PAPILLEDEMA-YES	5 (25%)
Differentiation Syndrome (DS) – Yes	14 (70%)
Differentiation Syndrome (DS) – No	6 (30%)
DS Grade II	6 (30%)
DS Grade III	8 (40%)

Temporary discontinuation of study medications was observed in six out of 20 patients (30%). The remaining 14 (70%) patients continued therapy without interruption. The discontinuation of ATRA in five (25%) patients was prompted by the development of papilledema, indicative of increased intracranial pressure, a known adverse event associated with retinoid therapy. ATO was temporarily withheld in one patient (1, 5%) due to transaminitis, reflecting hepatic toxicity. In the single case where both agents were withheld, the clinical presentation involved a combination of adverse effects necessitating temporary cessation of therapy (Table 4).

**Table 4:** Temporary Discontinuation of Drugs ATRA: all-trans retinoic acid; ATO: arsenic trioxide

Parameter	Frequency (n=20)	Percentage (%)
Temporary Discontinuation of Drugs		
Yes	6	30%
No	14	70%
Type of Drugs Discontinued		
ATRA only	5	25%
ATO only	1	5%
Both ATRA and ATO	1	5%

After the induction phase, 13 (68.42%) patients were MRD-positive, and six (31.58%) patients were MRD-negative. By the consolidation phase, all patients were MRD-negative (19, 100%). In terms of the final outcome, the majority of patients were alive (19, 95%), with one death (1, 5%), attributed to intracranial bleeding during induction (Table 5).

**Table 5:** Clinical Response and Final Outcome MRD: minimum residual disease

Parameter	Value
MRD Status After Induction Phase	
Negative	6 (31.58%)
Positive	13 (68.42%)
MRD Status After Consolidation Phase	
Negative	19 (100%)
Positive	0
Outcomes	
Alive	19 (95%)
Death (due to intracranial bleeding during induction)	1 (5%)

At the end of the study, only one (5.26%) patient had deranged lipid levels or triglyceridemia, while 18 (94.74%) patients had normal lipid profiles (Table 6).

**Table 6:** Prevalence of Deranged Lipid Profile

Status	n (%)
Yes	1 (5.26%)
No	18 (94.74%)

**Figure 2:**
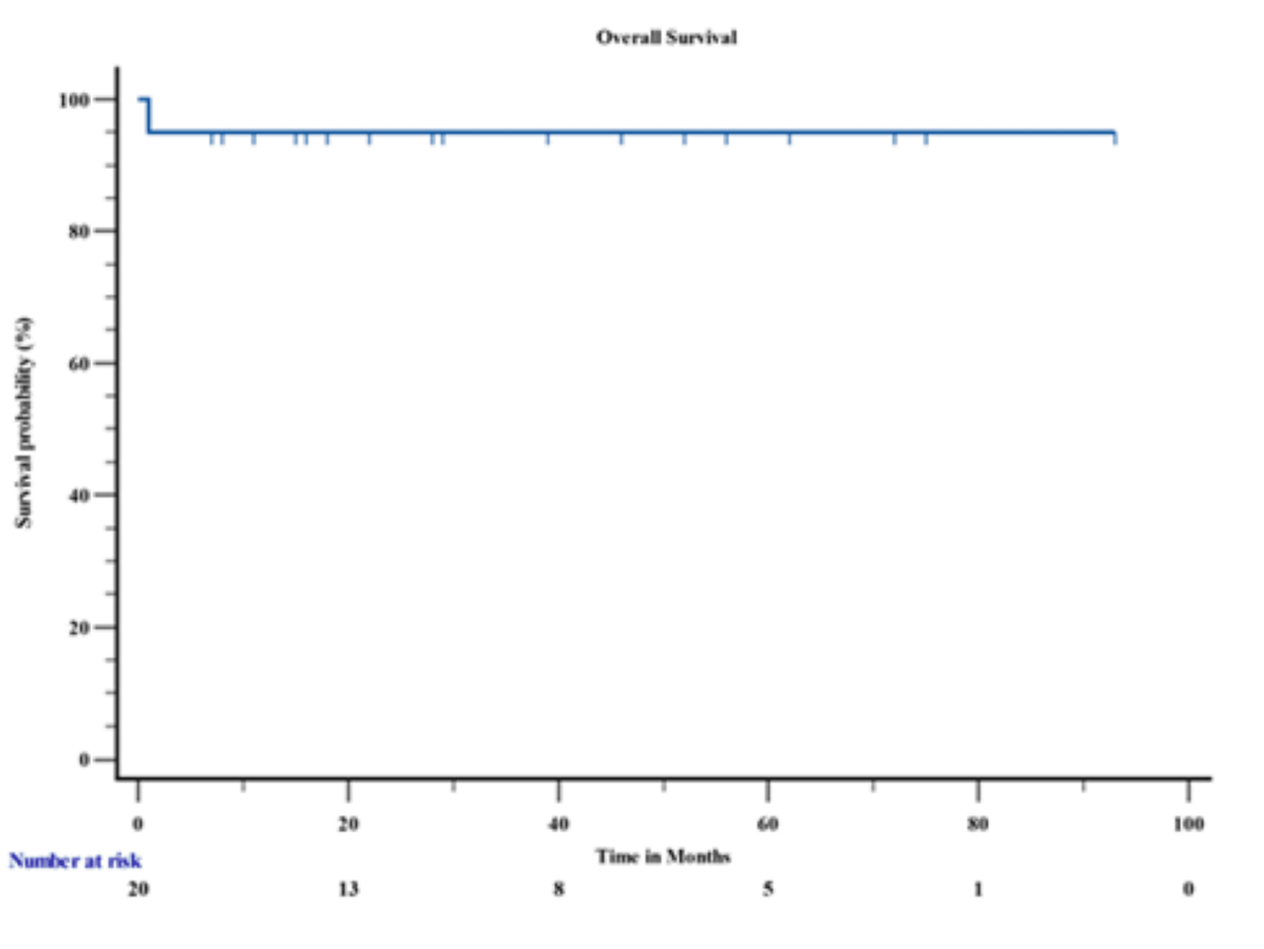
Overall Survival Analysis

Out of the 20 patients, 19 (95%) completed the induction therapy and achieved complete remission, only one (5%) patient died during induction therapy leading to an OS of 88 months (95% CI= 79.612 to 97.188) (Figure 2).

**Figure 3:**
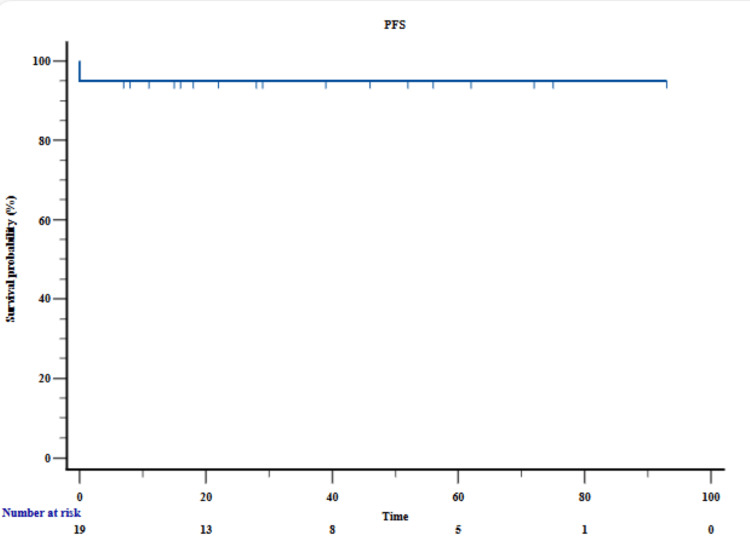
Progression free survival (PFS) analysis

The three-year PFS was 88 months (95% CI= 79.612 to 97.188). For standard-risk (SR) and high-risk (HR) patients, the three-year OS was 98% ± 3% and 86% ± 12%, respectively (Figure 3).

## Discussion

APL, while one of the most treatable subtypes of AML, presents significant challenges, particularly in resource-constrained settings [16]. The present study offers important insights into the clinical spectrum, therapeutic response, and survival outcomes of APL in a real-world, resource-constrained setting. A key strength of this cohort is the use of a chemotherapy-free regimen comprising ATRA and ATO. This approach eliminated the need for conventional cytotoxic chemotherapy, thereby reducing associated toxicities while maintaining excellent therapeutic efficacy. Notably, no relapses were observed in any of the patients during the follow-up period, highlighting the durability of remission with this regimen. The OS in our cohort was 88 months and the PFS was 88 months, which is a promising result given the aggressive nature of APL.

APL patients commonly exhibit severe bleeding diathesis, with approximately 90% experiencing symptoms ranging from mild mucocutaneous bleeding (such as gum bleeding and bruising) to life-threatening hemorrhages, often due to thrombocytopenia and coagulopathy leading to DIC [17]. Additionally, symptoms of pancytopenia, including fatigue and pallor from anemia, increased susceptibility to infections from neutropenia, and easy bruising from thrombocytopenia, are prevalent [18]. Clinically, most patients in the study presented with mucocutaneous symptoms (19, 95%) and bleeding manifestations (17, 85%), including gum bleeding (10, 50%) and purpura/ecchymosis (10, 50%). Pallor was observed in 19 (95%) patients, further emphasizing the severe anemia and bone marrow failure characteristic of this disease [4].

The diagnosis of APL is confirmed through molecular testing for the PML-RARα fusion gene and characteristic findings in blood smears [19]. In this study, the laboratory findings showed that most patients had moderate to severe anemia (mean hemoglobin level: 6.51 ± 1.82 mg/dL) and low platelet counts (mean platelets: 17.60 ± 19.10 × 10⁹/L), highlighting the significant hematologic abnormalities associated with APL. As is typical for this disease, the PML-RARA fusion transcript was markedly elevated (5175.95 ± 3039.37%), confirming the molecular diagnosis of APL. Coagulation abnormalities, including prolonged PT, elevated INR, increased D-dimer, and low fibrinogen levels, were common, indicating DIC, a hallmark of APL that contributes to its high morbidity and mortality.

Regarding the complications in the current study, febrile neutropenia was the most common adverse event, observed in 16 (80%) patients, followed by DS in 14 (70%) patients, with Grade II and Grade III DS seen in six (30%) and eight (40%), respectively. These complications are consistent with previous reports in the literature, where differentiation syndrome is a common complication in patients treated with ATRA and ATO (24.8%) [20]. Other complications, such as transaminitis, hyperbilirubinemia, and QTc prolongation, highlight the need for vigilant monitoring during therapy, particularly in high-risk patients.

Multisystem involvement is common during APL treatment and requires a multidisciplinary approach to manage. In a review by Abedin et al. (2016), Hemorrhagic complications (90%) were observed to be the leading cause of early mortality, particularly in high-risk patients (WBC >10 x 109/L). DS (20-25%) presented with fever, edema, and respiratory distress, with a higher risk in patients with elevated leukocyte counts. Coagulopathy, including DIC, was prevalent and required aggressive management. Infections due to neutropenia were common, while ATO-related QT interval prolongation posed a risk of cardiac arrhythmias. Relapse occurred in 10-20% of patients [21]. The absence of relapse in our study cohort is particularly significant in this context, demonstrating that a chemotherapy-free ATRA+ATO approach can offer not only high initial response rates but also sustained disease control.

In terms of lipid profile changes, we observed that only one (5.26%) patient developed deranged lipid levels or triglyceridemia by the end of the study, which is relatively low compared to a study by Sun et al. (2020) reporting higher incidences of lipid abnormalities in ATRA-based therapies (55.8%) [22]. The low incidence in our cohort may reflect differences in patient populations, treatment regimens, or supportive care practices. In our study, mortality was primarily attributed to complications arising during induction therapy, including coagulopathy and multiorgan dysfunction, which led to one death (5%). Importantly, this single fatality occurred during the induction phase, highlighting the urgent need for early detection and prompt management of life-threatening complications, especially coagulopathy. Similar results were observed by Karim et al. (2014), where 61.5% (16 out of 26) patients died during the induction period [23].

Strengths and limitations

This study offers real-world data on pediatric APL treated in a resource-limited setting. It includes detailed clinical profiles and presents long-term survival data in months, which is rarely reported in pediatric APL studies. The absence of relapse adds to its clinical relevance.

This study is limited by its retrospective nature and the small sample size of 20 patients, which may affect the generalizability of the findings. However, the results are significant for this region, providing valuable insights into the clinical course of APL in North India and suggesting that ATRA- and ATO-based regimens can lead to favorable outcomes in both SR and HR groups when managed appropriately.

## Conclusions

This study reinforces that chemotherapy-free regimens using ATRA and ATO are highly effective in the management of pediatric APL. The absence of relapses and the favorable long-term survival outcomes observed in this cohort are especially encouraging. While the overall toxicity profile was manageable, induction-related complications including febrile neutropenia, transaminitis, and QTc prolongation, highlights the importance of vigilant monitoring and proactive supportive care. Early diagnosis and timely initiation of therapy remain critical to improving survival outcomes and minimizing treatment-related morbidity. Going forward, incorporating standardized supportive care protocols and early risk stratification may further optimize outcomes and should be considered in routine clinical practice.
